# Role of the Environment Polarity on the Photophysical Properties of Mesogenic Hetero-Polymetallic Complexes

**DOI:** 10.3390/molecules29040750

**Published:** 2024-02-06

**Authors:** Adelina A. Andelescu, Angela Candreva, Evelyn Popa, Alexandru Visan, Carmen Cretu, Massimo La Deda, Elisabeta I. Szerb

**Affiliations:** 1 “Coriolan Drăgulescu” Institute of Chemistry, Romanian Academy, 24 Mihai Viteazu Bvd., 300223 Timisoara, Romania; aandelescu@acad-icht.tm.edu.ro (A.A.A.); pevelyn@acad-icht.tm.edu.ro (E.P.); visan.alexandru@acad-icht.tm.edu.ro (A.V.); cretucarmen@acad-icht.tm.edu.ro (C.C.); 2 Department of Chemistry and Chemical Technologies, University of Calabria, 87036 Rende, Italy; angela.candreva@unical.it; 3 Institute of Nanotechnology (NANOTEC), National Research Council (CNR), UOS Cosenza, 87036 Rende, Italy

**Keywords:** hetero-polymetallic coordination complexes, metallomesogens, emissive materials

## Abstract

New hetero-polynuclear coordination complexes based on a pentacoordinated Zn(II) metal center with tridentate terpyridine-based ligands and monoanionic gallates functionalized with long alkyl chains containing ferrocene units were designed, synthesized and characterized using spectroscopic and analytical methods. The complexes are mesomorphic, exhibiting columnar hexagonal mesophases. The photophysical properties in a solution and in an ordered condensed state were accurately investigated and the influence of the polarity of the solvent was evidenced.

## 1. Introduction

Emissive materials containing cheap biocompatible and bioavailable *d*-block metals like copper and zinc are considered promising in several applications, such as in optoelectronic devices [1,2,3], sensing [4,5,6], multi-stimuli responsive materials [7,8,9,10,11] and so on. The interest arises from the possibility of harvesting triplet excited states resulting in an exponential increase in emissions when compared with organic low-molecular-weight luminophores. For use in optoelectronic devices, the possibility of having luminophores capable of forming liquid-crystalline phases presents a great advantage, due to the possibility of forming films and regulating their properties through the application of electric fields [11,12].

Luminescent compounds in solution can undergo important changes in their photophysical properties when they form condensed phases, such as mesophases; in particular, these variations can be attributed to aggregation phenomena (which involve the formation of excimers or actual stacked systems that profoundly modify the emissive electronic states) [12] and/or to the variation in environmental polarity passing from the solution phase to the condensed one. Generally, this last aspect is rather neglected when the photophysics of mesogenic molecules is studied and compared with that observed in solution.

2,2′:6′,2″-terpyridine (*tpy*) and its derivatives are important chelating ligands in coordination and supramolecular chemistry, yielding complexes with excellent electronic, optical and electrochemical properties [13,14,15,16,17,18,19,20]. Their coordination complexes, based on the cheap biocompatible and bioavailable Zn(II) metal center, were successfully used for obtaining polarized emissions [21] and emissive materials for optoelectronics and luminescence-based technologies [22,23], sensing and detection [24,25,26,27,28,29,30,31,32,33], DNA binding and/or cleavage agents [34,35,36,37] and bioimaging [38,39,40].

Increasing the complexity of the molecular structure by introducing a second metal center and increasing nuclearity may induce new synergistic properties in the photophysical, electrochemical or sensing properties. The objective to be achieved was to insert a second metal center in the molecular structure of a mesomorphic Zn(II) complex already synthesized and characterized by us [21], maintaining the pentacoordination induced by the Zn(II) ion and to follow its influence on the mesogenic and photophysical properties of the final complex. This was achieved through the insertion of a second metal center using a ferrocene core into the aliphatic part of the molecule.

**Figure 1:**
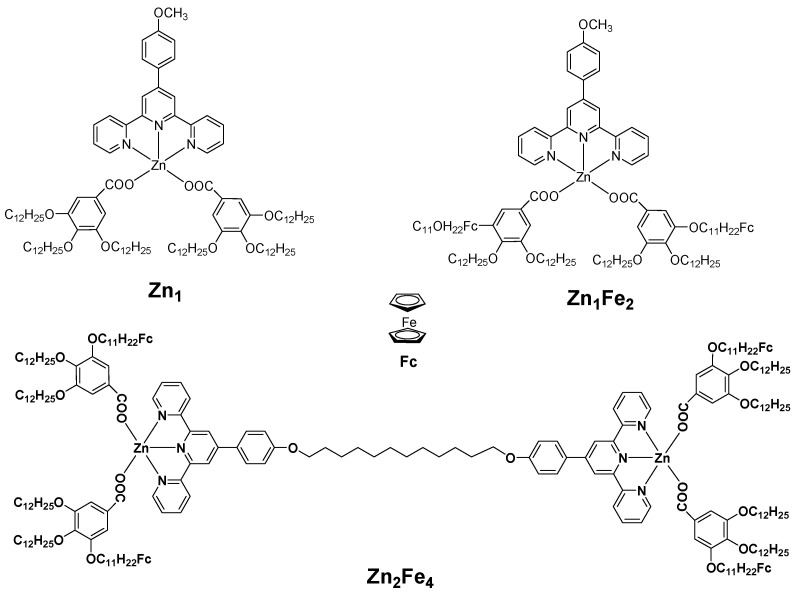
Molecular structure of Zn(II) *tpy*-based coordination complexes under study.

Hence, we synthesized complex Zn_1_Fe_2_ and, to double the nuclearity, Zn_2_Fe_4_ (see Figure 1), investigating their photophysical and mesogenic properties; the parent compound Zn_1_, previously described [21], is reported here for comparison.

## 2. Results

The complexes Zn_1_Fe_2_ and Zn_2_Fe_4_ were obtained following a synthetic strategy previously reported (see Section 4.1) [19]. Their structure and purity were determined using spectroscopic (FT-IR, ^1^H NMR), atomic absorption spectrometry and analytic (elemental analysis) methods, while the mesomorphic properties were assessed through polarized optical microscopy (POM) and differential scanning calorimetry (DSC) techniques. Emission maxima were followed in different polarity solutions and mixtures of solvents for determining the influence of supramolecular structures and molecular environments on the photophysical properties. Moreover, for the Zn_2_Fe_4_, the photophysical properties in the ordered phase were also conducted.

### 2.1. Synthesis and Structural Characterisation

**Scheme 1:**
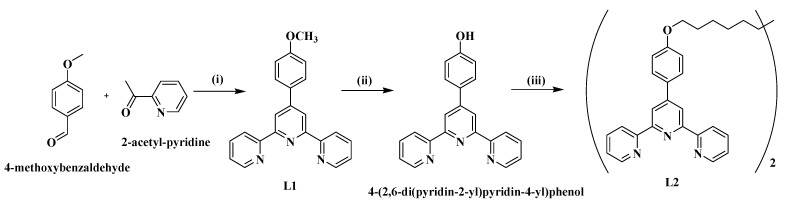
Synthetic pathway for L1 and L2. Conditions: (i) KOH, NH_4_OH, EtOH, ΔT, 24 h; (ii) CH_3_COOH/HBr, ΔT, 24 h; (iii) 1,12-dibromododecane, K_2_CO_3_, DMF, 80 °C, 24 h.

**Scheme 2:**
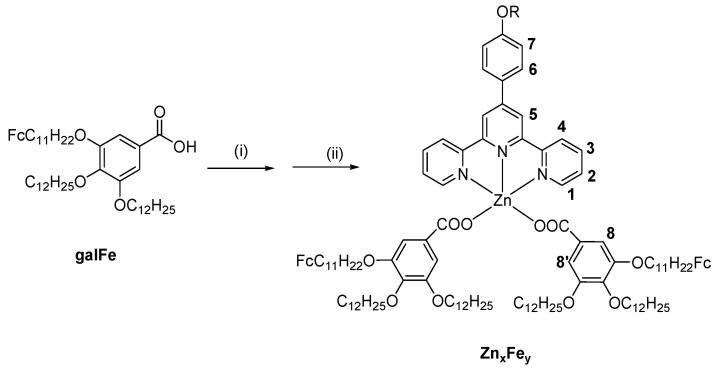
Synthetic pathway for complexes ZnFe_2_ and Zn_2_Fe_4_. Conditions: (i) NaOH, 30 min, EtOH, then ZnCl_2_ in EtOH, room temperature, 24 h; (ii) L_n_, CHCl_3_, 24 h.

The synthesis of the ligands and complexes was carried out following methods described in the literature (Scheme 1 and Scheme 2) [19,41,42].

In particular, ligand L1 was obtained by a one-pot Kröhnke condensation between 4-methoxybenzaldehyde and 2-acetylpyridine, in a basic media (See Section 4.1) [41,42]. Ligand L2 was obtained from L1 after demethylation to the hydroxyl form (4-(2,6-di(pyridin-2-yl)pyridin-4-yl)phenol) under strong acidic conditions and the isolation of this intermediate, followed by a Williamson etherification procedure with 1,12-dibromododecane in a 2:1 molar ratio (See Section 4.1).

The synthesis of the ancillary gallate ligands containing ferrocene units (galFe) was described previously [19]. The final complexes were obtained in a two-step reaction, involving the synthesis of a Zn-gallate precursor that was reacted with the corresponding *tpy* ligand (Scheme 2—Section 4.1).

**Figure 2:**
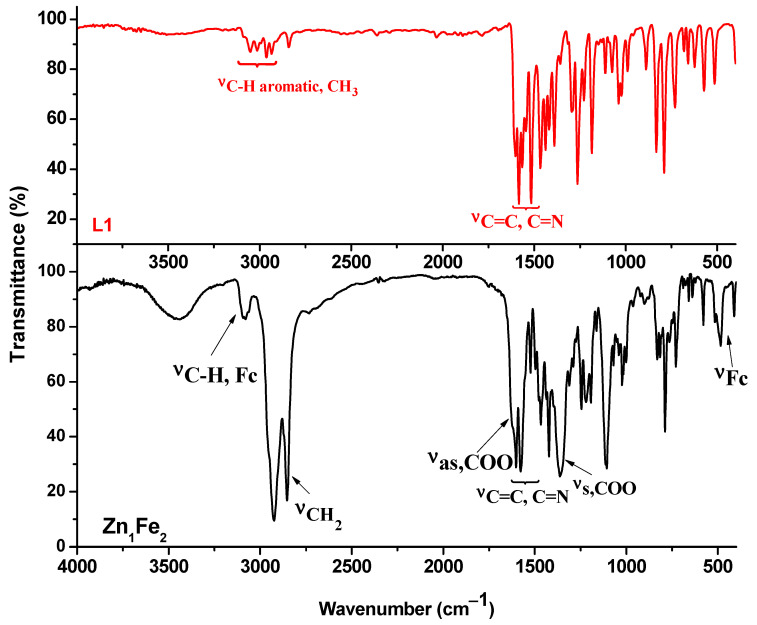
FT-IR spectra of ligand L1 and complex Zn_1_Fe_2_ with the principal absorption bands evidenced.

As for the analogues Zn(II) complexes previously reported [19,21], the pentacoordination around Zn is fulfilled by a *tpy*-chelating center and two monodentate units of gallate derivative. The successful coordination was firstly assessed using FT-IR spectroscopy, by the significant shift to higher frequencies of the ν_C=C_ and ν_C=N_ ligand characteristic bands (Figure 2 and Figure S3—Supplementary Information) and the monodentate coordination mode of the gallate units evidenced by the separation of the stretching vibrations of the COO^−^ group: Δ ≈ 260 cm^−1^ [43]. The shift in the aromatic protons of the *tpy* ligand close to the coordination center and the singlet due to the gallate aromatic proton observed in the ^1^H NMR spectra further confirmed the obtainment of the coordination complexes (Figures S1 and S2—Supplementary Information). Moreover, the purity of the final complexes was assessed using elemental analysis and atomic absorption spectrometry (AAS—Section 4.1).

### 2.2. Mesomorphic Properties

**Figure 3:**
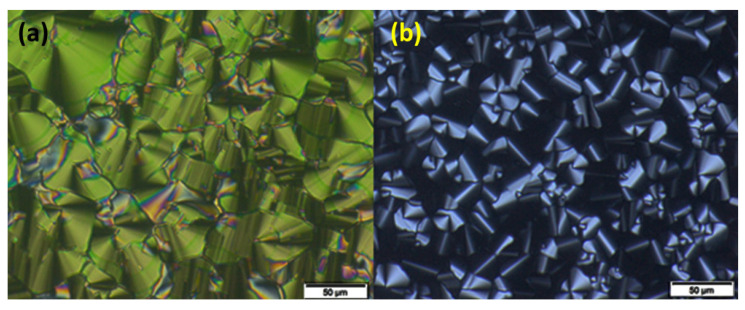
POM micrograph of the textures developed by (**a**) Zn_1_Fe_2_ on first cooling, at 30 °C, magnification 20× and (**b**) Zn_2_Fe_4_ on first cooling, at 100 °C, magnification 20×.

The typical fan-shaped focal textures with homeotropic zones observed by POM (Figure 3) suggests that the coordination complexes herein synthesized probably self-assemble into columnar hexagonal liquid crystalline mesophases, which exhibit these common features [44].

**Figure 4:**
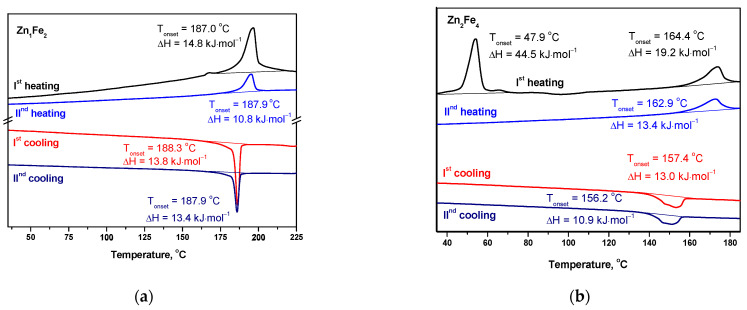
DSC thermograms of (**a**) complex Zn_1_Fe_2_ and (**b**) complex Zn_2_Fe_4_.

**Figure 5:**
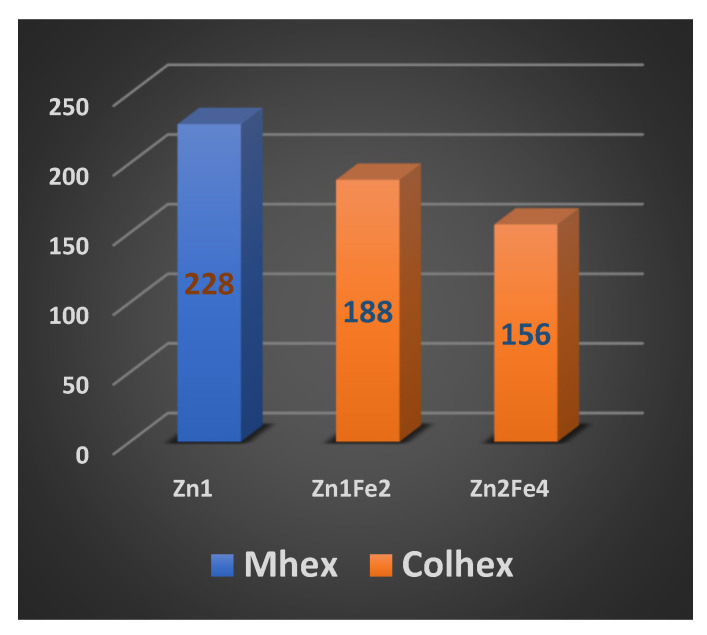
Bar graph with the mesomorphic properties of complexes.

The introduction of the ferrocene unit in the liquid-like region of the columnar structure decreases the transition temperatures and the order degree, similar to the parent compound previously reported [19]. Indeed, while complex Zn_1_ arranges into a three-dimensional columnar hexagonal mesophase (M_hex_), from room temperature to 229 °C [21], complexes Zn_1_Fe_2_ and Zn_2_Fe_4_ likely self-assemble into classical 2D Col_hex_, with lower isotropisation temperatures (Figure 4 and Figure 5), in a similar way to its parent complex, as previously reported [19]. While complex Zn_1_Fe_2_ is mesomorphic, from room temperature to 187 °C, complex Zn_2_Fe_4_ has a transition close to room temperature only on the first heating cycle, indicating a Cr to mesophase transformation. However, on the cooling cycle, no transition is observed, with the order in the mesophase being frozen at room temperature.

### 2.3. Photophysics

**Figure 6:**
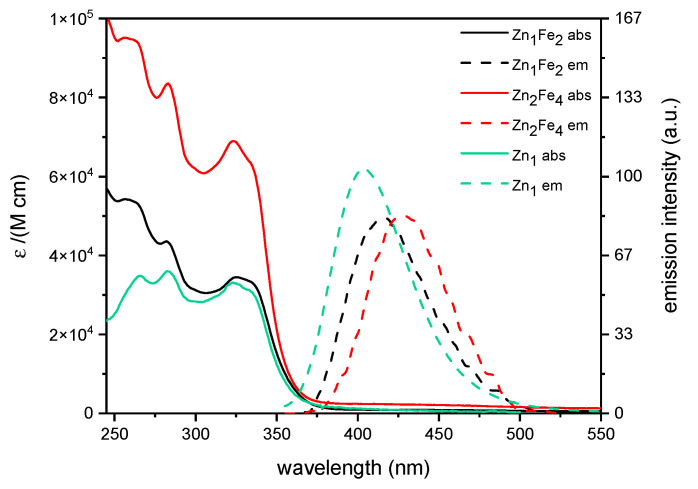
Absorption and emission spectra of 1.0 × 10^−5^ M dichloromethane solution of the complexes.

The photophysical properties of the complexes were first recorded from dilute dichloromethane solutions (see Figure 6).

The absorption spectra are very similar: they all have three main bands each accompanied by shoulders. The first two bands, positioned at 260 and 283 nm, respectively, are attributed to ligand-centered (LC) transitions located on the terpyridine fragment (see Figures S4–S7—Supplementary Information) [21], while the third band, positioned at 325 nm, is attributed to a ligand-to-ligand charge transfer (LLCT) transition from the gallate to the terpyridine ligand [43]. All complexes are fluorescent, showing a band maximum ranging from 405 to 428 nm, depending on the complex (405 nm for Zn_1_; 414 nm for Zn_1_Fe_2_; 428 nm for Zn_2_Fe_4_), but all are due to the deactivation from LLCT state. The change in the position of the emission maximum is related to the increase in the Stokes shift going from Zn_1_ to Zn_1_Fe_2_ to Zn_2_Fe_4_. In fact, while the absorption spectrum of the three complexes is superimposable, the Stokes shift increases from 6208 cm^−1^ to 6806 cm^−1^ to 7650 cm^−1^, respectively. This is due to the increase in vibrational motions induced by the introduction of the Fc cores (Zn_1_Fe_2_) and the aliphatic chain that connects the two fractions (Zn_2_Fe_4_).

**Figure 7:**
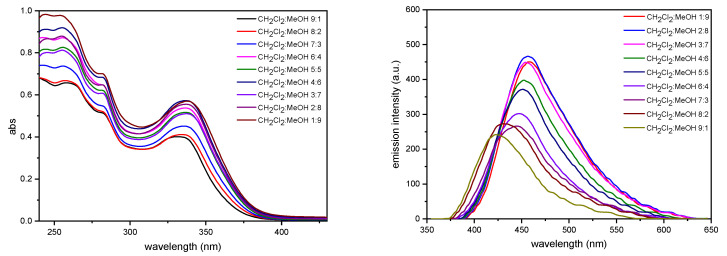
Absorption (**left**) and emission (**right**) spectra of Zn_2_Fe_4_ in 1.0 × 10^−5^ M dichloromethane solution recorded at increasing amounts of methanol.

To explore the effect of the polarity of the environment on the photophysical properties, a stock solution of Zn_2_Fe_4_ 1.0 × 10^−4^ M was prepared in dichloromethane solution. The stock solution was used to prepare 10 probes of 1.0 × 10^−5^ M in mixtures of solvents. To keep concentration and volume constant, subsequent dilutions were carried out with proper volumes of methanol and dichloromethane. The dilutions were made according to Table S1, as reported in the Supplementary Information. Absorption and emission spectra are reported in Figure 7.

The absorption bands, considering the experimental uncertainty, show a constant trend: as the methanol fraction increases, their intensity increases. The emission band instead undergoes a bathochromic shift as the percentage of methanol increases, while the emission intensity increases as the alcoholic fraction increases, especially when this becomes greater than 50%.

To verify whether these variations could depend on the concentration of the solutions, the absorption and emission spectra were collected in the two pure solvents at different concentrations, but only a linear growth of the spectral intensities in concentration was observed (spectra not reported).

**Figure 8:**
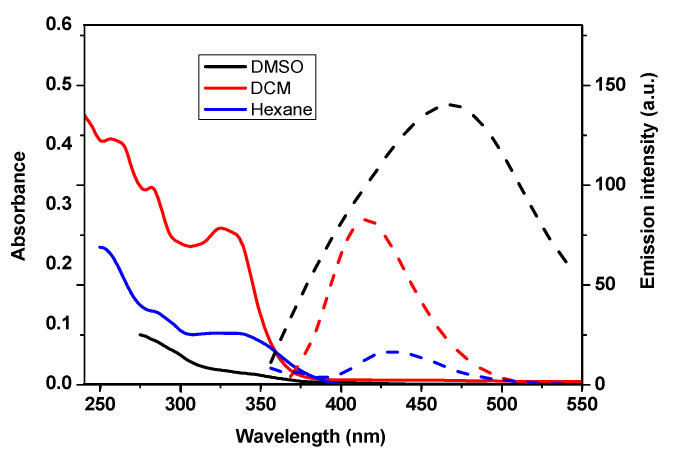
Absorption solid line) and emission (dash line) spectra of Zn_2_Fe_4_ in 1.0 × 10^−5^ M in solvents with different polarities.

**Figure 9:**
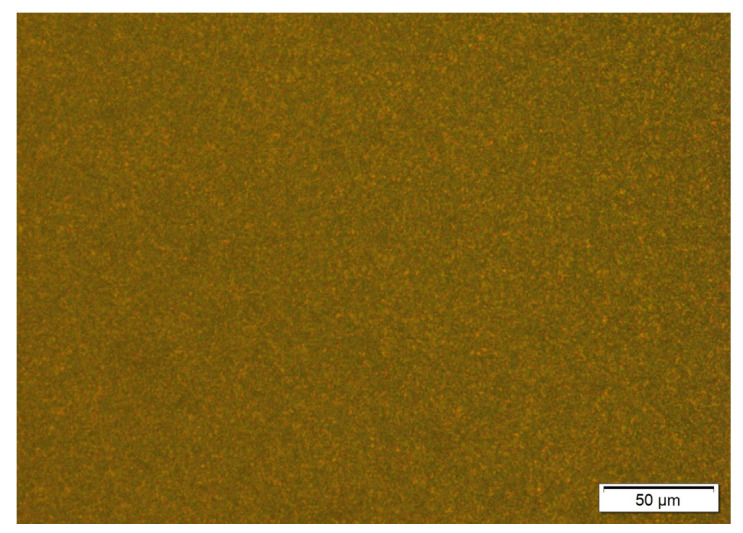
POM micrograph of the Zn_2_Fe_4_ film thermally treated: birefringent texture indicates liquid crystalline order.

The photophysical properties of Zn_2_Fe_4_ were determined in solvents with different polarities: more polar DMSO and less polar hexane, respectively (See Figure 8). In both cases, however, hypochromic shifts of the absorption maxima and the red-shift of the emission maxima were observed. The photophysical properties of Zn_2_Fe_4_ were also determined in the mesophase by obtaining a film drop casted from a concentrated DCM solution on a glass plate. The solvent was evaporated, the film was dried under vacuum and thermally treated by heating it until the isotropic phase and then slowly cooled down. The order was checked by POM (Figure 9).

**Figure 10:**
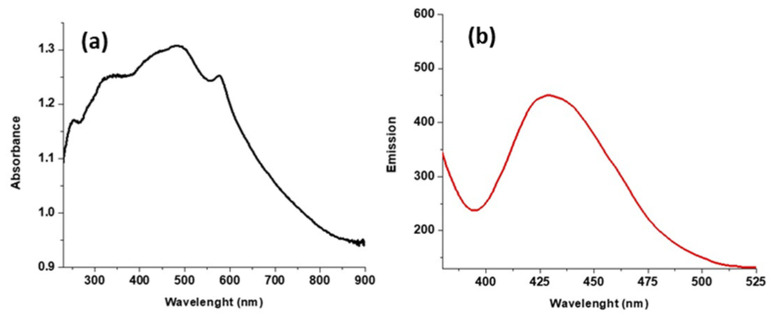
(**a**) Absorption and (**b**) emission (excited at 350 nm) of the ordered Zn_2_Fe_4_ film.

The absorption spectrum in mesophase is reported in Figure 10a. Compared with the absorption spectrum recorded in the DCM solution, it shows additional bands at 490 and 580 nm due to the formation of the supramolecular mesogenic structure, which leads to an energy lowering of the electronic states compared with the isolated molecule. These electronic states are primarily non-radiatively deactivated, because mesophase is not luminescent if excited on these electronic levels. However, by exciting mesophase samples at 350 nm (i.e., on the levels attributed to the isolate molecules), an emission band peaking at 430 nm (Figure 10b), attributable through a comparison of the solution sample (Figure 7) with the isolated molecule, has been recorded. This behavior seems to indicate, in the mesophase structure, the different surroundings of the molecules, causing the presence of molecules which, while not iterating significantly, show an emission identical to that of the isolated molecule, while, at smaller distances, they give rise to π–π stacking interactions, which do not present emissions, but are responsible for the absorption bands around 580 nm.

## 3. Discussion

The introduction of a second metal center in the aliphatic regions of complex Zn_1_ increased the symmetry of the mesophase and decreased transition temperatures, as expected when compared with the parent compound reported previously [19]. From a photophysical point of view, the introduction of the Fc core does not modify absorption or emission features; Fc absorption bands fall in the UV part of the spectrum, so they are superimposable to the gallate and terpyridine bands, while Fc is not emissive, and the distance of the Fc core from the fluorophoric centers of ZnFe_2_ and Zn_2_Fe_4_ preserves their luminescence. However, in condensed “soft” phases, the properties of the complexes depend not only on the molecular structure but are also influenced by the supramolecular arrangements in the mesophase and the molecular environment. Hence, the influence of the polarity of the media were studied in solution.

The variation in the emission intensity as the polarity of the solvent varies, in principle, can be due to various non-mutually exclusive factors: the formation of aggregates (due to a different solubility in the new solvent), and the effect of the refractive index of the solvent; finally, it is necessary to consider the possibility that by modifying the solvent, the specific interactions of the fluorophore with the solvent can be established, for example the formation of hydrogen bonds, acid-base chemistry or charge transfer interactions [45]. The formation of aggregates as methanol is added can be excluded since, if Zn_2_Fe_4_ was prone to form aggregates that modify its photophysics, such structures should also form as the concentration increases, but no such changes in absorption or luminescence were observed as the concentration of the complex varied in either methanol or dichloromethane. The absence of variations in the shape of the absorption and emission spectra allows us to exclude the presence of specific solvent/complex interactions. Based on this, the increase in the intensity of the absorption and emission spectrum and the red-shift of the luminescence that are observed with the increase in the alcohol fraction can be attributed to the increase in the polarity of the surrounding environment.

When passing to the condensed liquid crystalline state from the solution, the absorption properties change significantly, leading to the formation of lower energy states. These states are not emissive, the quenching of the emissions being attributable to the formation of non-emissive aggregates but also to the change in the polarity of the molecular surroundings. However, some emissions arising from the presence of “isolated” molecules can be detected, similar to the emissions in the DCM solution.

## 4. Materials and Methods

All precursor materials and solvents were commercially available and were used as received without further purification.

2-acetylpyridine 99%, 4-methoxybenzaldehyde, sodium hydroxide (NaOH), potassium hydroxide (KOH), potassium carbonate (K_2_CO_3_), 1,12-dibromododecane, zinc chloride (ZnCl_2_) and hydrobromic acid 48% (HBr) were purchased from Sigma Aldrich (Burlington, MA, USA). Glacial acetic acid (CH_3_COOH), ethanol (EtOH), ammonium hydroxide 25% (NH_4_OH), and ammonium chloride (NH_4_Cl) were from Chimreactiv (Bucuresti*,* Romania). *N*,*N*-Dimethylformamide (DMF), dichloromethane (DCM) and methanol (MeOH) were purchased from Honeywell (Tokyo, Japan), whereas chloroform (CHCl_3_) and hexane were from Carlo Erba (Cornaredo, Italy).

Infrared spectra (KBr) were recorded using a Cary (Agilent, Penang, Malaysia) 630 FT-IR spectrophotometer in the range 4000–400 cm^−1^ in transmittance.

A Bruker Fourier (Wissembourg, France) 80 Research FT-NMR Benchtop 80 MHz spectrometer was used to record ^1^H NMR experiments in CDCl_3_ for precursors, while for ligands and final coordination complexes, a Bruker (Billerica, MA, USA) NEO 400 MHz spectrometer was used. Elemental analysis was carried out using a Flash (Pune, India) 2000 microanalyser from Thermo Fisher Scientific (Waltham, MA, USA). Zn(II) % was determined using a Sens AA flame atomic absorption spectrometer (GBC Scientific Equipment, Braeside, Australia) equipped with a zinc hollow cathode lamp. The detection limit was 0.4–1.5 mg/L and the integration time 3 s in air–acetylene mixture. An average absorbance value of two determinations was reported.

Optical textures of the mesophases were observed with an Olympus (Tokyo, Japan) BX53M polarizing microscope (POM) equipped with a Linkam hot stage and an Olympus UC90 camera. DSC traces were recorded with a Q1000 apparatus from TA Instruments (New Castle, DE, USA) calibrated with indium; three heating/cooling cycles were performed for each sample, with a heating and cooling rate of 10 °C/min.

Spectrofluorimetric grade solvents were used for the photophysical investigations in solution, at room temperature. An Agilent Cary (Santa Clara, CA, USA) 60 spectrophotometer was employed to obtain the absorption spectra, while the corrected emission spectra, all confirmed by excitation ones, were recorded with a on a Perkin-Elmer Model LS 55 apparatus (PerkinElmer, Inc./UK Model LS 55, Waltham, MA, USA), using 1 cm path length cells. 

Electronic spectra in mesophase of complex Zn_2_Fe_4_ were recorded using a JASCO V-650 double-beam spectrophotometer (Tokyo, Japan) with a photomultiplier tube detector, 60 mm integrating sphere coated with barium sulfate.

### 4.1. Experimental Section

#### 4.1.1. Synthesis of Ligands

L1: In a one-neck round-bottom flask, 2-acetylpyridine (12.1 g, 99.08 mmol), 4-methoxybenzaldehyde (6.8 g, 49.94 mmol) and KOH (8.5 g, 151.5 mmol) were dissolved in EtOH (150 mL), then the reaction mixture was cooled at 0 °C in an ice bath and stirred for 30 min at this temperature. Then, NH_4_OH 25% (75 mL) was added dropwise. During the addition of ammonia, a formation of a precipitate was noticed. The reaction mixture was allowed to reach room temperature, a condenser was connected and the stirring was continued for 24 h at reflux (cca 50 °C). After cooling to room temperature, the crystalline precipitate was filtered off and washed with cold methanol, yielding a white solid (10.3 g, 30.3 mmol, 60%). 

^1^H-NMR (400 MHz, CDCl_3_, δ-ppm): 8.85–8.56 (overlapped peaks, 6H), 7.95–7.75 (overlapped peaks, 4H), 7.36 (ddd, *J* = 7.5, 4.8, 1.2 Hz, 2H), 7.05 (d, *J* = 8.8 Hz, 2H), 3.90 (s, 3H).

4-(2,6-di(pyridin-2-yl)pyridin-4-yl)phenol: Ligand L1 (10.9 g, 32.1 mmol) was refluxed in a mixture of 48% HBr (10 mL) and glacial CH_3_COOH (10 mL) for 24 h, then cooled to room temperature and neutralized with NaOH 20%. The precipitate was filtered off and washed thoroughly with water, resulting in a pale grey solid (10.21 g, 31.3 mmol, 95%).

^1^H-NMR (400 MHz, DMSO-d_6,_ δ-ppm): 8.78–8.70 (d, 2H), 8.68–8.59 (d, 4H), 8.07–7.95 (td, 2H), 7.82–7.61 (m, 2H), 7.58–7.43 (m, 2H), 7.00–6.95 (dd, 2H). 

L2: A mixture of 4-(2,6-di(pyridin-2-yl)pyridin-4-yl)phenol (2.00 g, 6.147 mmol) and K_2_CO_3_ (2.55 g, 18.441 mmol) in 50 mL DMF was heated to 65 °C and stirred at this temperature for 30 min, then 1,12-dibromododecane (1.01 g, 3.078 mmol) in 10 mL DMF was added to the mixture. The reaction mixture was stirred at 80 °C for 24 h, cooled to room temperature and filtered. The mixture was diluted with water (300 mL) and extracted with DCM (4 × 100 mL). The combined organic layers were washed with H_2_O (5 × 100 mL), NH_4_Cl (100 mL) saturated and NaCl saturated (100 mL). Recrystallization from CH_2_Cl_2_/MeOH afforded the pure product as a white powder (1.70 g, 2.09 mmol, 68%).

^1^H-NMR (400 MHz, CDCl_3,_ δ- ppm): 8.65 (overlapped peaks, 12H), 7.78 (overlapped peaks, 8H), 7.30 (t, *J* = 7.8 Hz, 4H), 7.05 (d, *J* = 8.5 Hz, 4H), 3.94 (t, *J* = 6.1 Hz, 4H), 1.85 (m, 4H), 1.30 (m, 16H).

#### 4.1.2. Synthesis of Complexes

To a solution of galFe [19] (0.200 g, 0.237 mmol) in 15 mL CHCl_3_ was added dropwise an ethanolic solution of NaOH (0.0095 g, 0.237 mmol). The reaction mixture was stirred at room temperature for 30 min, then ZnCl_2_ (0.016 g, 0.119 mmol), dissolved in the minimum amount of EtOH, was added dropwise. After 60 min of stirring, the solvent was removed under reduced pressure to give a pale orange residue, which was used without any purifications in the next step.

The residue was dissolved in 10 mL of CHCl_3_, and a solution of ligand Ln (0.119 mmol L1/0.0595 mmol L2) in 10 mL CHCl_3_ was added dropwise resulting in a brown solution which was stirred at r.t for 24 h. After that, the solvents were removed under reduced pressure. The complexes were obtained as yellow solids, as following: Zn_1_Fe_2_: recrystallisation from acetone (64%). Anal. Calcd. for C_126_H_183_Fe_2_N_3_O_11_Zn (2092.89 g·mol^−1^): C, 72.31; H, 8.81; N, 2.01. Found: C, 72.40; H, 8.79; N, 2.02%. AAS: Zn% calcd.: 3.12, found: 3.15. ^1^H NMR (400 MHz, CDCl_3_, δ/ppm) 8.97 (d, *J* = 4.9 Hz, 2H, H^1^), 8.27 (s, 2H, H^5^), 8.12 (d, *J* = 8.1 Hz, 2H, H^4^), 7.71 (overlapped peaks, 4H, H^6,3^), 7.35 (overlapped peaks, 2H, H^2,8,8′^), 6.81 (d, *J* = 8.7 Hz, 2H, H^7^), 4.03 (overlapped peaks, 33H, -OC*H*_2_-, -OC*H*_3_, H^Fc^), 2.32 (m, 4H, -C*H*_2_-Fc), 1.75–1.10 (overlapped peaks, 122 H), 0.92 (t, *J* = 4.9 Hz, 12H).

FT-IR (KBr, cm^−1^): ν _C-H, Fc_ (3080), ν_CH2,as_ (2924), ν_CH2,s_ (2863), 1625 (ν_as COO_-), 1360 (ν_s COO_-): Δ = 265 cm^−1^, ν_C=O_ (1578), ν_C-O_ (1245, 1106), out of-plane vibration of Fc cyclopentadiene (1002, 914), ν_ring-Fe,Fc_ (486).

Zn_2_Fe_4_: recrystallisation hexane/EtOH (70%). Anal. Calcd. for C_262_H_384_Fe_4_N_6_O_22_Zn_2_ (4324.04 g·mol^−1^): C, 72.77; H, 8.95; N, 1.94. Found: C, 72.71; H, 9.01; N, 1.95%. AAS: Zn% calcd.: 3.02, found: 3.03. ^1^H NMR (400 MHz, CDCl_3_, δ/ppm) 8.91 (d, ^3^*J* = 4.6, 4H, H^1^), 8.20 (overlapped peaks, 8H, H^5,4^), 7.64 (overlapped peaks, 8H, H^6,3^), 7.28 (overlapped peaks, 12H, H^2,8,8′^), 6.76 (d, ^3^*J* = 8.3 Hz, 4H, H^7^), 3.96 (overlapped peaks, 64 H, OC*H*_2_, H^Fc^), 2.20 (m, 8H, -C*H*_2_-Fc), 1.75–1.10 (overlapped peaks, 252 H), 0.85 (t, *J* = 4.9 Hz, 24H).

FT-IR (KBr, cm^−1^): ν_C-H,Fc_ (3091), ν_CH2,as_ (2924), ν_CH2,s_ (2863), 1620 (ν_as COO_-), 1362 (ν_s COO_-): Δ = 258 cm^−1^, ν_C=O_ (1594), ν_C-O_ (1218, 1114), out of-plane vibration of Fc cyclopentadiene (1002, 902), ν_ring-Fe,Fc_ (483).

## 5. Conclusions

In search of emissive “soft” materials, the synthesis of new hetero-polymetallic liquid-crystalline coordination complexes was carried out. The complexes resulted as mesomorphic at room temperature or close to room temperature, exhibiting column hexagonal phases in a large temperature range. The study of the photophysical properties of liquid-crystalline compounds starts from their characterization in solution. However, before comparing the behavior of these compounds in solution and in mesophase, it is necessary to study the effect of the environment on photophysics. In the liquid-crystalline phase, in fact, intermolecular interactions become important, but sometimes the effects of the change in polarity of the environment are neglected. In this work, we wanted to conduct a preliminary study of the photophysics of a hetero-polymetallic mesogenic compound by varying the polarity of the solvent, and observing how an increase in polarity leads to an increase in emissions and their bathochromic shift, in the absence of the formation of aggregates that could influence photophysics. However, by the formation of aggregates, the quenching of the emissions in the mesophase was obtained and only a weak emission arising from “isolated” molecules, which are probably not forming the columnar structures, was observed. By synthesizing hetero-polynuclear coordination complexes, new and synergistic properties can be expected. However, the design of highly luminescent metallomesogens has to consider several factors that influence the emission properties in condensed states including the polarity of the molecular environment.

## Data Availability

The data presented in this study are available in article and Supplementary Materials.

## Supplementary Materials

The following supporting information can be downloaded at: https://www.mdpi.com/article/10.3390/molecules29040750/s1, Figure S1: ^1^H NMR spectra of ligand L1 and complex Zn_1_Fe_2_ in CDCl_3_; Figure S2: ^1^H NMR spectra of ligand L2 and complex Zn_2_Fe_4_ in CDCl_3_; Figure S3: FT-IR spectra of ligand L2 and complex Zn_2_Fe_4_; Table S1: Method preparation of the samples used for the investigation of the effect of the polarity of the environment; Figure S4: Absorption spectrum of L1 in dichloromethane solution; Figure S5: Emission spectrum of L1 in dichloromethane solution; Figure S6: Absorption spectrum of L2 in dichloromethane solution; Figure S7: Emission spectrum of L2 in dichloromethane solution.
