# Supplementary material for: Role of the Environment Polarity on the Photophysical Properties of Mesogenic Hetero-Polymetallic Complexes
Source: Molecules. 2024 Feb 6;29(4):750. doi: 10.3390/molecules29040750 (PMC10893215; doi:10.3390/molecules29040750)
Supplement: Supplementary file 1 [file molecules-29-00750-s001.zip › molecules-2774934-supplementary.pdf]

SUPPLIMENTARY INFORMATION

for

# Role of the Environment Polarity on the Photophysical Properties of Mesogenic Hetero-Polymetallic Complexes

**Adelina A. Andelescu [1], Angela Candreva [2,3], Evelyn Popa [1], Alexandru Visan [1], Carmen Cretu [1], Massimo La Deda [2,3,*] and Elisabeta I. Szerb [1,*]**

[1] "Coriolan Drăgulescu" Institute of Chemistry, Romanian Academy, 24 Mihai Viteazu Bvd., 300223 Timisoara, Romania; aandelescu@acad-icht.tm.edu.ro (A.A.A.); pevelyn@acad-icht.tm.edu.ro (E.P.); visan.alexandru@acad-icht.tm.edu.ro (A.V.); cretucarmen@acad-icht.tm.edu.ro (C.C.)

[2] Department of Chemistry and Chemical Technologies, University of Calabria, 87036 Rende, Italy; angela.candreva@unical.it

[3] Institute of Nanotechnology (NANOTEC), National Research Council (CNR), UOS Cosenza, 87036 Rende, Italy

* Correspondence: massimo.ladeda@unical.it (M.L.D.); eszerb@acad-icht.tm.edu.ro (E.I.S.)

$^1$H NMR spectra of ligand **L1** and complex **Zn$_1$Fe$_2$** in $CDCl_3$. **Figure S1**……………………………………2

$^1$H NMR spectra of ligand **L2** and complex **Zn$_2$Fe$_4$** in $CDCl_3$. **Figure S2**……………………………………3

FT-IR spectra of ligand **L2** and complex **Zn$_2$Fe$_4$. Figure S3**……………………………………………4

Method preparation of the samples used for the investigation of the effect of the polarity of the environment. **Table S1.** ……………………………………………………………………………………5

Absorption spectrum of **L1** in dichloromethane solution. **Figure S4**………………………………………… 6

Emission spectrum of **L1** in dichloromethane solution. **Figure S5**……………………………………………..6

Absorption spectrum of **L2** in dichloromethane solution. **Figure S6**…………………………………………..7

Emission spectrum of **L2** in dichloromethane solution. **Figure S7**…………………………………..………7

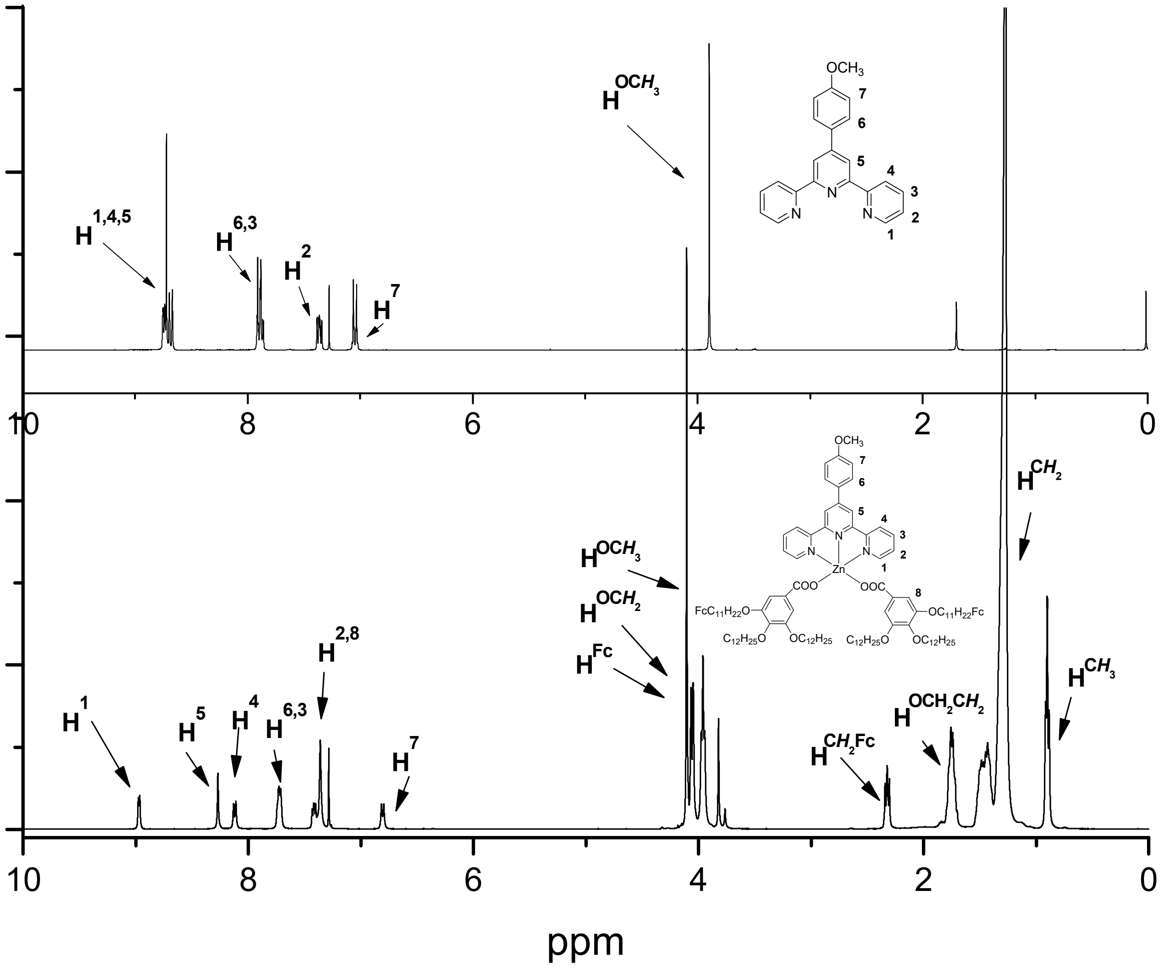


**Figure S1**. $^1$H NMR spectra of ligand L1 and complex $Zn_1Fe_2$ in $CDCl_3$.

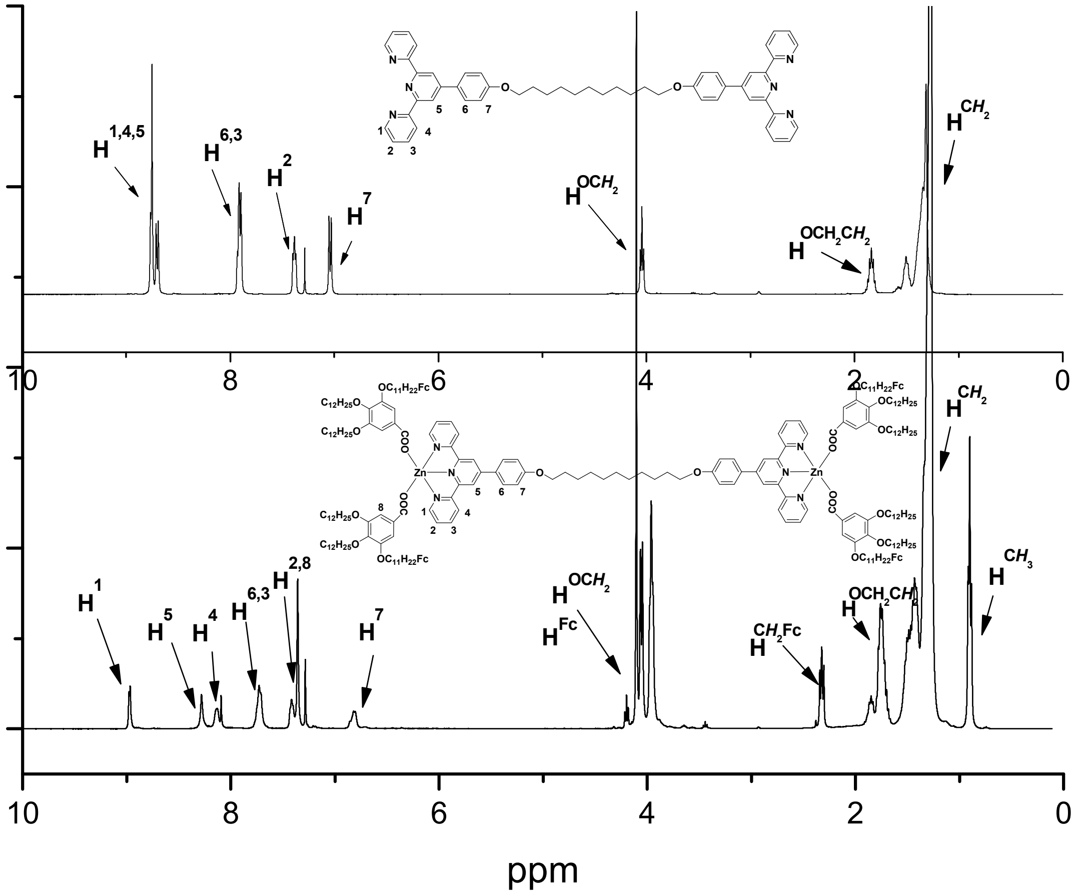

**Figure S2**. $^{1}H$ NMR spectra of ligand L2 and complex $Zn_2Fe_4$ in $CDCl_3$.

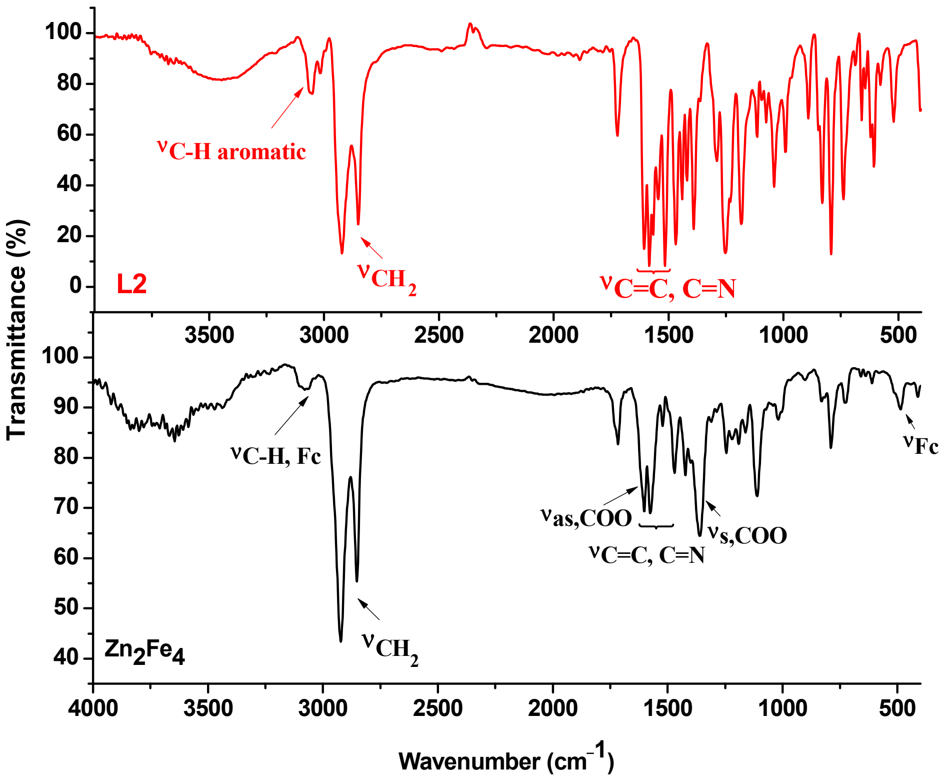


**Figure S3**. FT-IR spectra of ligand L2 and complex $Zn_2Fe_4$ with the principal absorption bands evidenced.

**Table S1.** Method preparation of the samples used for the investigation of the effect of the polarity of the environment.

| Stock solution of 1.0 $E^{-4}$ M in dichloromethane | Dichloromethane | Methanol | Volume of the final solution | Final concentration | % Methanol |
|---|---|---|---|---|---|
| 1 mL | 9 mL | 0 mL | 10 mL | 1.0 $E^{-5}$ M | 0 |
| 1 mL | 8 mL | 1 mL | 10 mL | 1.0 $E^{-5}$ M | 10 |
| 1 mL | 7 mL | 2 mL | 10 mL | 1.0 $E^{-5}$ M | 20 |
| 1 mL | 6 mL | 3 mL | 10 mL | 1.0 $E^{-5}$ M | 30 |
| 1 mL | 5 mL | 4 mL | 10 mL | 1.0 $E^{-5}$ M | 40 |
| 1 mL | 4 mL | 5 mL | 10 mL | 1.0 $E^{-5}$ M | 50 |
| 1 mL | 3 mL | 6 mL | 10 mL | 1.0 $E^{-5}$ M | 60 |
| 1 mL | 2 mL | 7 mL | 10 mL | 1.0 $E^{-5}$ M | 70 |
| 1 mL | 1 mL | 8 mL | 10 mL | 1.0 $E^{-5}$ M | 80 |
| 1 mL | 0 mL | 9 mL | 10 mL | 1.0 $E^{-5}$ M | 90 |

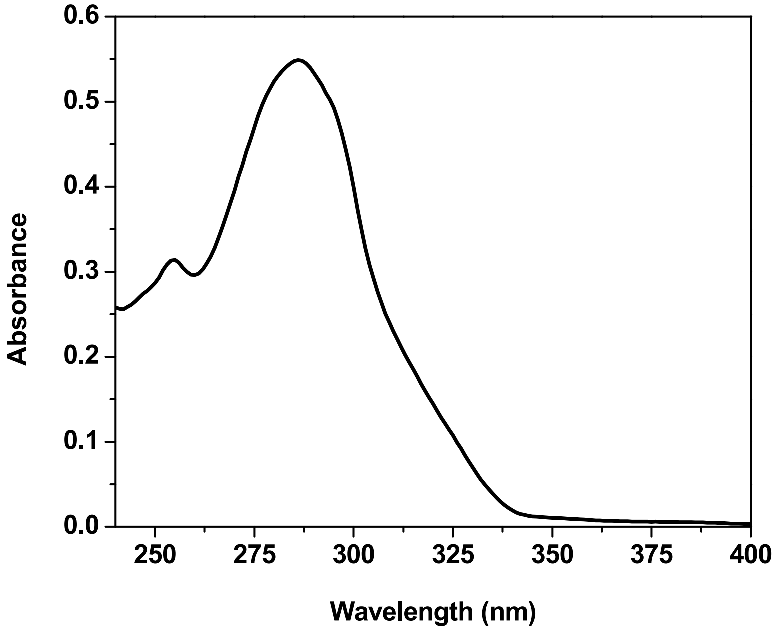


**Figure S4**. Absorption spectrum of L1 in 1.0 × $10^{-5}$ M dichloromethane solution (light path = 1 cm)

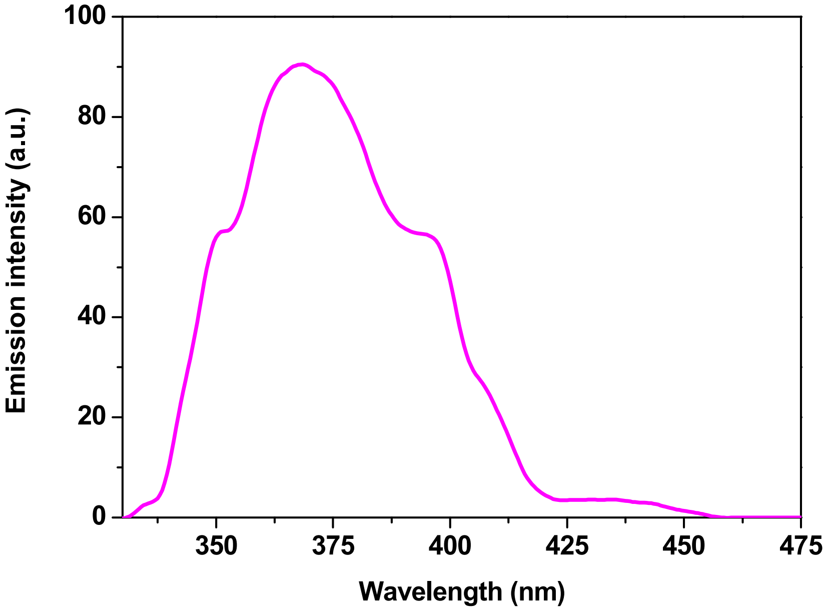


**Figure S5**. Emission spectrum of L1 in dichloromethane solution.

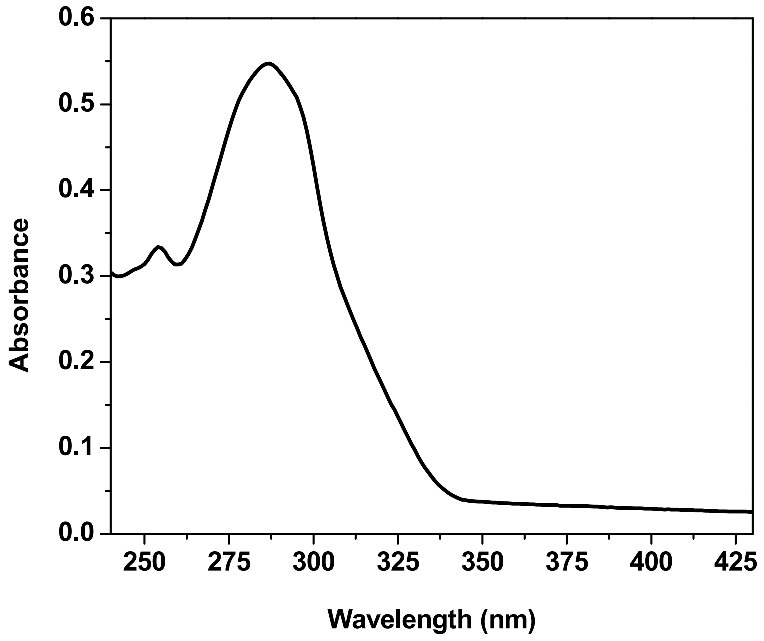


**Figure S6**. Absorption spectrum of L2 in 1.0 × $10^{-5}$ M dichloromethane solution (light path = 1 cm).

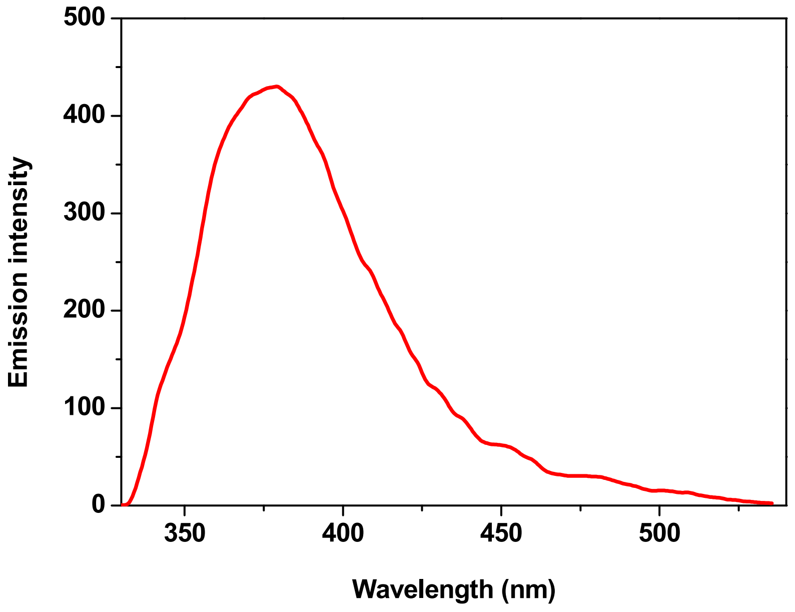


**Figure S7**. Emission spectrum of L2 in dichloromethane solution.
